# Towards molecular diagnostics of parental alienation

**DOI:** 10.1007/s00018-025-05895-3

**Published:** 2025-11-06

**Authors:** Oleksandr Kamyshnyi, Iryna Kamyshna, Pavlo Petakh, Iryna Halabitska, Magnar Bjoras, Valentyn Oksenych, Marie-Pierre Schoving, Denis E. Kainov

**Affiliations:** 1 Department of Microbiology, Virology, and Immunology, I. Horbachevsky Ternopil National Medical University, Ternopil, 46001 Ukraine; 2 Department of Medical Rehabilitation, I. Horbachevsky Ternopil National Medical University, Ternopil, 46001 Ukraine; 3 Department of Biochemistry and Pharmacology, Uzhhorod National University, Uzhhorod, Ukraine; 4 Department of Therapy and Family Medicine, I. Horbachevsky Ternopil National Medical University, Ternopil, 46001 Ukraine; 5 Department of Clinical and Molecular Medicine, Norwegian University of Science and Technology, Trondheim, Norway; 6 Department of Microbiology, Oslo University Hospital and University of Oslo, Oslo, Norway; 7 Centre of Healthy Embryology (CRESCO), Oslo, Norway; 8 Department of Child Psychiatry, Pitié-Salpêtrière, Paris, France

**Keywords:** Parental alienation, Chronic stress, Molecular diagnostics, Child health, HPA axis dysregulation

## Abstract

High-conflict parental divorce can result in parental alienation (PA), leading to chronic psychological stress in children that adversely affects their mental and physical health. This stress may manifest as anxiety, depression, eating disorders, immune dysfunction, and cardiovascular, neurological, endocrine, or gastrointestinal issues. The inconsistent legal recognition of PA highlights the need for objective diagnostic tools to inform judicial decisions. We propose a panel of molecular biomarkers, derived from well-established indicators of chronic stress, to address the limited data specific to PA. This panel targets hypothalamic-pituitary-adrenal (HPA) axis activation, neurotransmitter dysregulation, inflammation, oxidative stress, epigenetic modifications, and gut microbiota dysbiosis. Interdisciplinary collaboration among researchers, general practitioners, and legal professionals is crucial to validate and implement this panel, ultimately enhancing child welfare through early intervention.

**Supplementary Information:**

The online version contains supplementary material available at 10.1007/s00018-025-05895-3.

## Introduction

Parental divorce is a prevalent phenomenon, affecting approximately 40–50% of marriages in the United States, impacting over 1 million children annually [1]. When accompanied by high conflict, divorce can lead to child alienation, where one parent manipulates the child to reject the other [2]. Estimates suggest over 22 million U.S. adults have experienced PA, with 10 million facing severe alienation [2]. More conservative estimates suggest 11–15% of divorces involving children globally may include PA [3].

Parental divorce introduces significant stress, particularly in high-conflict scenarios, affecting many children who do not live with both biological parents [3]. PA exacerbates this stress, leading to feelings of isolation, anger, and low self-esteem [4]. The mental health impact, including anxiety, depression, suicidal ideation, and trauma reactions, is supported by direct evidence from studies which report increased psychopathological symptoms [5], and identify long-term emotional consequences in adults alienated as children [5]. Adults who experienced PA as children often face emotional issues, such as anxiety or thoughts of self-harm, and struggle with relationships [6].

Parental alienation syndrome (PAS) was first described by Dr. Gardner in 1985 [7], but its diagnostic status remains controversial. The most recent DSM-5-TR and ICD-11 do not list PAS as a separate disorder; instead, alienating behavior is coded under broader “parent–child relationship problems” (DSM-5-TR Z62.820; ICD-11 QE52). Whether PAS deserves its own entry is still under debate. Supporters argue that an official label would help clinicians detect cases early and allow courts to intervene more decisively, whereas critics note that the research base is still small, existing studies suffer sampling bias, and the label could obscure genuine abuse [8, 9].

Stress stemming from intense parental conflict and alienation can precipitate a wide spectrum of health problems—from eating disorders and immune dysfunction to cardiovascular risk, neurocognitive impairment, endocrine disturbance, and gastrointestinal dysfunction [10]. Because longitudinal, biomarker-based studies in alienated children are still rare, mechanistic insights must often be extrapolated from other well-characterized models of chronic pediatric stress (e.g., academic overload, bereavement, chronic illness) [11]. Those models nevertheless point to measurable shifts in hormones, inflammatory mediators, epigenetic marks, oxidative-stress products, and gut-microbiota profiles that could serve as diagnostic or prognostic biomarkers of PA.

This review therefore argues for the development of a molecular test panel that translates those biomarkers into an objective read-out of stress load in alienated children. Such a tool would empower general practitioners, especially GPs, to detect physiological harm early, tailor clinical interventions, and provide clear evidence for legal proceedings aimed at mitigating ongoing abuse.

### Legal issues of PA

From a legal perspective, PA poses significant challenges in family court, where it is often raised in custody disputes. Family law attorneys and judges must carefully evaluate allegations of alienation, as these claims can be manipulated to gain leverage in custody battles, potentially overshadowing the child’s best interests. Courts typically prioritize the child’s welfare, guided by legal standards such as the “best interests of the child” doctrine, which considers factors like parental fitness, emotional bonds, and the child’s health and safety. However, the lack of a universally accepted definition of PA complicates judicial decisions. Some jurisdictions recognize alienation as a form of psychological abuse, potentially justifying modifications to custody arrangements or court-ordered interventions like reunification therapy. Others remain skeptical, citing insufficient empirical evidence to classify it as a diagnosable syndrome [9]. Legal professionals must balance these considerations with evidence from health experts, such as psychologists or GPs, to assess the child’s emotional and physical health. Missteps in legal proceedings, such as failing to address alienation or wrongly attributing a child’s rejection of a parent to abuse, can exacerbate stress and health issues, underscoring the need for interdisciplinary collaboration between legal and medical professionals. The controversy surrounding PA as a syndrome, with critics arguing it lacks robust empirical support, highlights the need for better clinical approaches [9].

### Clinical issues of PA

To identify PA, clinicians use a model with five factors, including the child’s rejection of a parent without good cause and a history of a good relationship with that parent [8]. It should be noted that PA is different from cases where a child rejects a parent for valid reasons, like abuse.

Treating PA is challenging and requires careful steps. Clinicians focus on understanding family relationships to guide treatment [12, 13]. Family therapy or cognitive behavioral therapy helps, but there’s no standard treatment backed by strong evidence [14]. Rebuilding the relationship with the rejected parent takes time and often needs the child to take part willingly, with support from therapy. Clinicians need proper training to recognize PA and avoid mistakes that could worsen the situation. Some children who experience PA may later repeat the pattern as parents, passing it on to the next generation.

**Fig. 1:**
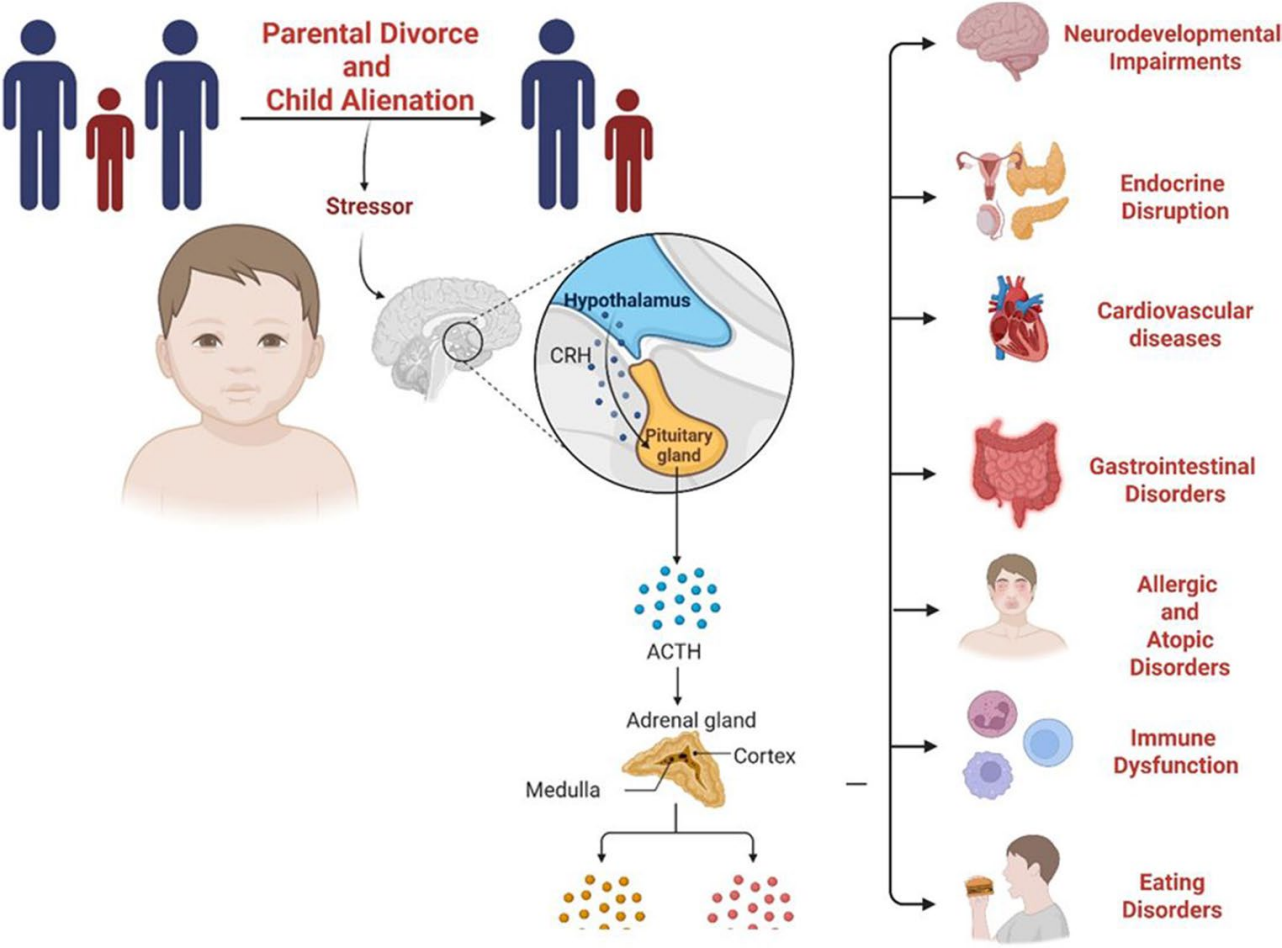
HPA axis activation pathway. Parental divorce and child alienation create emotional stress that activates the HPA axis. It begins with family conflict, affecting the hypothalamus and pituitary gland, which then signal the adrenal gland to produce stress hormones. This hormonal response contributes to a range of child health issues, including neurodevelopmental, immune, cardiovascular, and digestive problems

Untreated PA leads to a spectrum of health issues in alienated children (Table 1; Fig. 1). Only a handful of longitudinal investigations have followed alienated children specifically, yet those that exist show higher rates of major depressive disorder, generalized anxiety, oppositional defiant disorder, and substance misuse after high-conflict divorce [11–14]. To explain these outcomes, clinicians draw on a broader body of pediatric stress research derived from contexts such as academic overload, chronic illness, and bereavement [11].

**Table 1:** Molecular mechanisms and associated health impacts behind child alienation

Mechanism	Health impact
HPA axis dysregulation	Eating disorders, T1D, endocrine disruptions
Neurotransmitter imbalances	Eating disorders, neurological impairments
Inflammation	Immune dysfunction, allergies, cardiovascular issues
Oxidative stress	Cardiovascular, neurological damage
Epigenetic changes	Neurological, endocrine disruptions
Microbiota dysbiosis	Eating disorders, gastrointestinal issues, immunity

### Biological issues behind PA

**Fig. 2:**
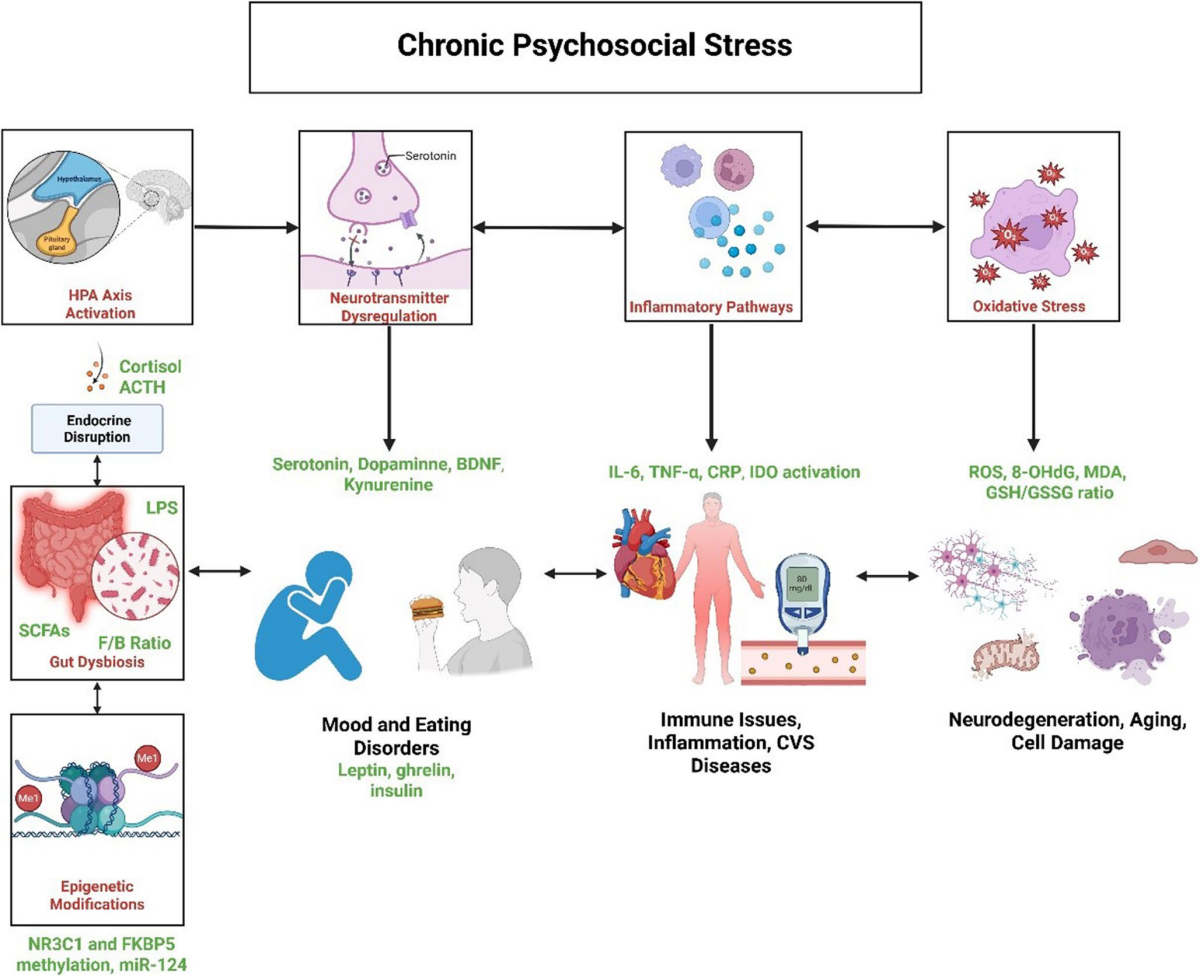
Potential biomarkers of PA. Chronic psychosocial stress arising from child alienation engages at least six tightly linked pathways: HPA‑axis activation (cortisol, ACTH); neurotransmitter dysregulation (serotonin, dopamine); inflammation (IL‑6, TNF‑α, CRP); oxidative stress (ROS, 8‑OHdG, MDA, GSH/GSSG ratio); regulation of gene expression (NR3C1 and FKBP5 methylation, miR‑124); gut dysbiosis (SCFAs, Firmicutes/Bacteroidetes ratio, LPS); and metabolic/endocrine shifts (leptin, ghrelin, insulin). Solid arrows depict experimentally verified feed‑forward effects, while dashed arrows represent feedback or crosstalk. The listed molecules are candidate biomarkers for a molecular test aimed at objectively identifying stress load in children experiencing parental alienation

Although some biological pathways underlying psychosocial stress are known, others remain unclear. These pathways initiate a cascade of molecular interactions that contribute to diseases triggered by PA. Mental health disorders, such as depression and anxiety, result from disruptions in neurotransmitter systems and the hypothalamic-pituitary-adrenal (HPA) axis. Eating disorders, including anorexia and bulimia, arise from metabolic dysregulation and gut-brain axis sequelae (see Table 2) [11, 14–19]. Immune dysfunction and allergies reflect inflammatory and gut dysbiosis effects, and metabolic risks (e.g., type 1 diabetes, hypertension) emerge from oxidative and autoimmune interactions. Neurological impairments and endocrine disruptions are tied to neurodevelopmental and epigenetic changes [11, 14–19]. Thus, HPA axis activation, neurotransmitter dysregulation, inflammation, oxidative stress, epigenetic modifications, and gut dysbiosis are keys to better understand PA and its consequences (Fig. 2).

**Table 2:** Eating disorders in divorced vs. intact families

MEBS subscale	Intact families (a^2^, e^2^)	Divorced families (a^2^, e^2^)	Model fit
Total score	0.62, 0.38	0.69, 0.30	Constrained AE
Weight preoccupation	0.53, 0.47	0.49, 0.51	Constrained AE
Body dissatisfaction	0.56, 0.44	0.76, 0.24	Unconstrained AE
Binge eating	0.36, 0.64	0.37, 0.63	Constrained AE

HPA axis activation initiates persistent cortisol surges (10- to 12-fold increases), disrupting glucose homeostasis, promoting visceral adiposity, and inducing insulin resistance, while blunting lymphocyte proliferation and pro-inflammatory feedback, increasing infection risk [15, 16]. This cortisol elevation feeds into neurotransmitter dysregulation, where excess glucocorticoids skew serotonin, dopamine, and norepinephrine signaling, laying a neural foundation for anxiety, depression, and addiction, and concurrently perturbing growth, thyroid, and gonadal hormones to delay somatic development [20–26].

The inflammatory pathways are both a consequence and driver of these effects. Cortisol-mediated immunosuppression is compounded by stress-induced rises in IL-6, TNF-α, and C-reactive protein (CRP), producing low-grade systemic inflammation linked to cardiovascular, metabolic, and autoimmune diseases. This inflammation amplifies HPA axis activity via cytokine-driven hypothalamic CRH release [27], creating a feed-forward loop. Sympathetic activation further boosts histamine and IgE responses, worsening asthma, rhinitis, and atopic dermatitis in alienated children [27–31].

Gut dysbiosis emerges as stress alters autonomic tone, intestinal permeability, and microbiota composition, reducing the Firmicutes/Bacteroidetes ratio and increasing lipopolysaccharide (LPS) [18, 19, 32, 33] (Table S1). This dysbiosis fuels irritable bowel and inflammatory bowel disorders and feeds back to mood circuitry, exacerbating neurotransmitter imbalances. The resultant inflammation further interacts with oxidative stress, where stress-driven reactive oxygen species (ROS) overwhelm antioxidant defenses, damaging lipids, proteins, and DNA, and shortening telomeres [34–36].

Oxidative stress, in turn, exacerbates inflammation and contributes to endothelial dysfunction, promoting early hypertension and atherosclerosis [23–26, 37].

Epigenetic modifications provide a long-term imprint, with DNA methylation (e.g., NR3C1) and histone shifts recalibrating stress-reactivity genes, embedding vulnerability to psychiatric and metabolic diseases [23–26, 37]. These epigenetic changes modulate HPA axis sensitivity, reinforcing its dysregulation [38], while oxidative stress-induced DNA damage triggers additional methylation events [39]. Simultaneously, microRNA shifts recalibrate stress reactivity genes, embedding long-term vulnerability to metabolic and psychiatric disease [23–26, 37].

The biological pathways interact dynamically, amplifying the health impacts of chronic stress in alienated children. Together, these interactions jeopardize virtually every organ system, from neurodevelopmental delays to cardiovascular risks. They also reveal actionable molecular signatures (hormones, cytokines, oxidative markers, epigenetic tags, and microbiota profiles) that could be assembled into diagnostic panels for early detection and targeted intervention in high-conflict divorce settings.

### Molecular diagnostic test for PA

Molecular diagnostic tests, based on the pathways outlined above, could provide quantifiable biomarkers to support judicial decisions. For example, elevated cortisol levels, which can be measured in saliva, blood, or hair samples. For instance, cortisol levels exceeding 10–12 times the baseline (e.g., > 300 nmol/L in saliva) may indicate sustained HPA axis dysregulation [40].

Neurotransmitter imbalances, such as reduced serotonin or elevated norepinephrine, can be assessed via cerebrospinal fluid or blood plasma analyses, reflecting mood and behavioral disruptions [41, 42]. Inflammatory markers like interleukin-6 (IL-6), tumor necrosis factor-alpha (TNF-α), and C-reactive protein (CRP) can be quantified through blood tests, with elevated levels (> 3 mg/L for CRP) indicating stress-induced inflammation [43]. Oxidative stress markers, such as 8-hydroxy-2’-deoxyguanosine (8-OHdG), can be measured in urine or blood, signaling cellular damage [44]. Epigenetic changes, particularly DNA methylation of the glucocorticoid receptor gene (NR3C1), can be analyzed using polymerase chain reaction (PCR) on blood samples, revealing stress-related gene expression alterations [45]. Gut microbiota dysbiosis, linked to gastrointestinal and mood disorders, can be evaluated through fecal 16 S rRNA sequencing to identify microbial imbalances (e.g., reduced Firmicutes/Bacteroidetes ratio) [46]. A panel combining these biomarkers, such as cortisol, inflammatory cytokines, oxidative stress markers, epigenetic profiles, and microbiota composition, could create a molecular signature of child alienation.

Alternatively, multiomics tools such as transcriptomics, metabolomics, epigenomics, and microbiomics can be employed to detect and monitor relevant biomarkers. Transcriptomics can identify altered gene expression profiles, such as those regulating HPA axis activity or inflammatory cytokines, providing insights into neuroendocrine and immune dysregulation. Metabolomics can detect shifts in cortisol, glucose, leptin, and ghrelin levels, reflecting metabolic imbalances and gut–brain axis disruptions linked to stress-induced appetite changes or dysbiosis. Epigenomics can assess DNA methylation and histone modifications, particularly at stress-response genes, to uncover long-term epigenetic imprinting that increases psychiatric and cardiometabolic risks. Microbiomics can profile gut microbiota alterations associated with stress-driven dysbiosis, which exacerbates mood and inflammatory disorders [47]. Integrating these omics approaches into diagnostic panels enables early detection of molecular signatures, such as cortisol surges, cytokine imbalances, oxidative stress markers, and microbial shifts, facilitating targeted interventions like CBT or mindfulness to mitigate the long-term health impacts of child alienation.

Such an approach would require standardized protocols and reference ranges tailored to children in high-conflict divorce settings. Validation through longitudinal studies is essential to ensure reliability and specificity. By providing objective data, these tests could aid courts in recognizing alienation as a form of psychological abuse, informing custody decisions, and prioritizing child welfare, while fostering collaboration between medical and legal professionals.

### Challenges, future directions, and the role of gps in molecular diagnostics of PA

The development and implementation of molecular diagnostic panels for PA face significant challenges. A primary challenge is the lack of longitudinal, biomarker-based studies specifically focused on alienated children, which limits the validation of proposed biomarkers like cortisol, inflammatory cytokines, or epigenetic markers [11]. Current evidence relies heavily on extrapolation from chronic stress models (e.g., academic overload, bereavement), which may not fully capture PA’s unique stress profile [11]. Establishing standardized protocols and age-specific reference ranges for children in high-conflict divorce settings is further complicated by ethical and logistical barriers, such as obtaining consent and accessing vulnerable populations. Additionally, translating multiomics approaches (e.g., transcriptomics, metabolomics, epigenomics) into clinical practice demands substantial investment in technology, training, and regulatory approval, posing practical hurdles [43, 45, 48].

Insights from related fields highlight these challenges while underscoring the potential of molecular diagnostics. For instance, studies on cortisol and CRP in workplace stress have struggled with biomarker specificity [43], while epigenetic marker research in trauma and PTSD required large-scale longitudinal cohorts for reliability [45]. Depression studies also reveal interindividual variability in inflammatory markers, necessitating personalized reference ranges [48]. These examples emphasize the need for robust, PA-specific studies to address sample size, heterogeneity, and biomarker validation.

Legal and cultural variability further complicates the adoption of molecular diagnostics. Recognition of PA varies globally, influencing demand for biomarker tests. In the U.S., some courts acknowledge PA in custody disputes, while others dismiss it as pseudoscience [9]. The UK’s Family Justice Council (2025) prioritizes domestic abuse over PA claims, and the United Nations has criticized PA as a pseudo-concept [9]. Jurisdictions that recognize PA may adopt diagnostics more readily, but inconsistent legal standards hinder standardization. Cross-jurisdictional studies are thus essential to validate and adapt these tools effectively.

**Fig. 3:**
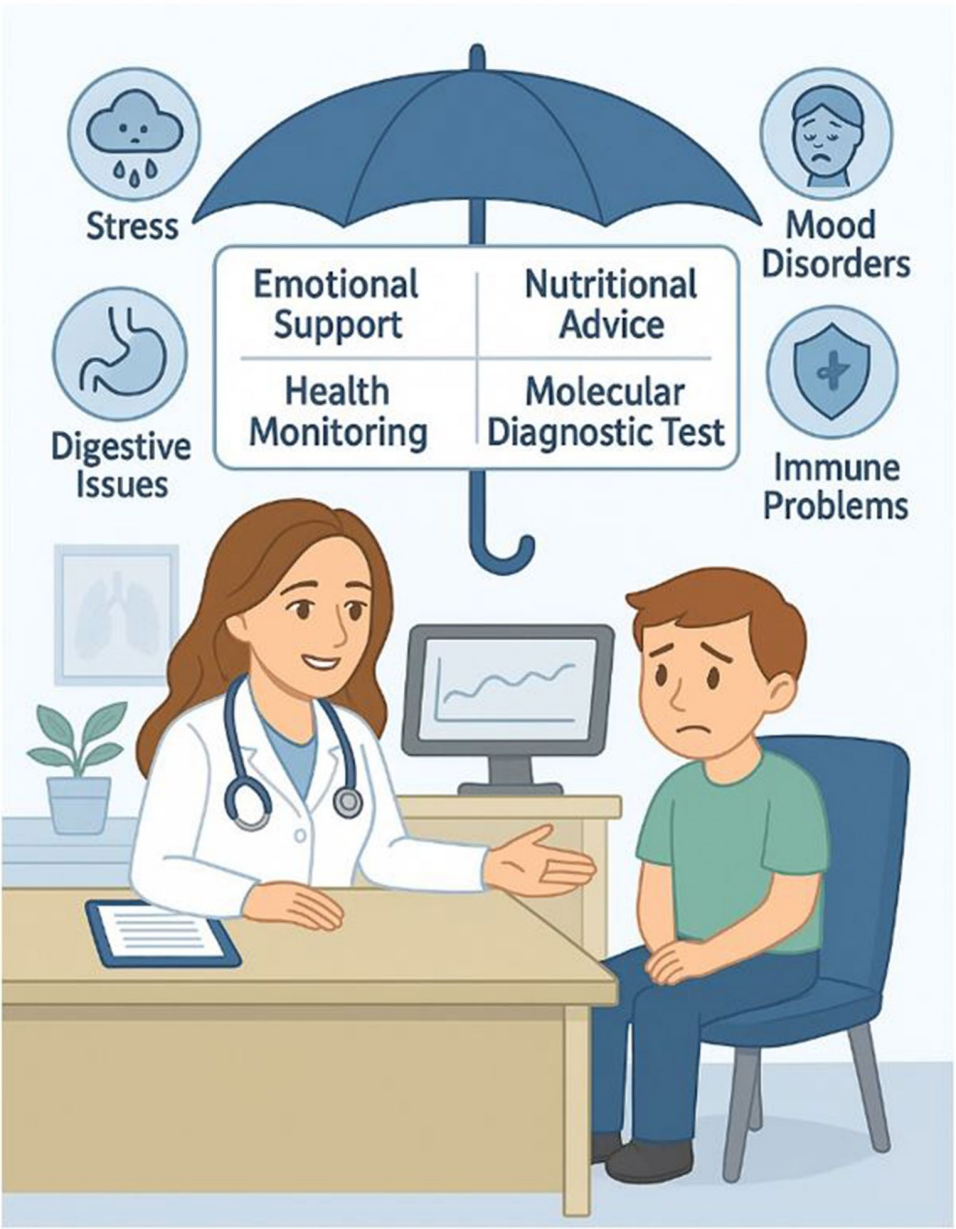
General practitioner’s role during parental divorce. GPs could refer child for diagnostic test, and provide emotional support, nutritional advice to a distressed child during parental divorce

GPs are uniquely positioned to address these challenges by serving as frontline healthcare providers in identifying and managing the biopsychosocial impacts of PA (Fig. 3) [49–54]. Their longitudinal relationships with families enable early detection of stress-related signs, such as anxiety (ICD-11: 6B00), depression (ICD-11: 6A70), eating disorders (ICD-11: 6B80), somatic complaints (e.g., headaches), and regressive behaviors, aligning with American Academy of Pediatrics guidelines [49, 50]. GPs can implement actionable steps, including routine monitoring using parental interviews or behavioral checklists, referring children for molecular diagnostic tests to assess physiological harm (e.g., elevated cortisol or inflammatory markers), and coordinating with psychologists or psychiatrists for therapies like cognitive behavioral therapy (CBT) when tests indicate risks of mental health issues. They can also connect families to family therapists, school counselors, or peer support groups to address relational dynamics.

Moreover, GPs play a critical role in interdisciplinary collaboration, supplying molecular test results and health assessments to legal professionals to inform custody decisions. In cases of suspected neglect, psychological abuse, or severe alienation, GPs should notify Child Protective Services (CPS), providing documented evidence (e.g., biomarker data, clinical observations) to protect child welfare [49, 53]. However, the variable legal recognition of PA across jurisdictions complicates this process, as courts may require robust, objective data to justify interventions like reunification therapy [9]. GPs can bridge this gap by advocating for the integration of molecular diagnostics into clinical and legal frameworks, ensuring holistic care that mitigates long-term developmental and psychosocial impacts.

Future directions include conducting longitudinal studies to validate PA-specific biomarkers, developing standardized testing protocols, and integrating multiomics data into accessible diagnostic tools. Collaboration among researchers, clinicians (particularly GPs), and legal professionals is essential to overcome these challenges, ensuring that molecular diagnostics can support early intervention and judicial decisions in high-conflict divorce cases, ultimately prioritizing child welfare.

## Conclusions

PA trigger chronic stress, driving health issues through different molecular mechanisms, though direct evidence from alienated children remains limited, relying on broader stress research. The proposed molecular diagnostic test, using a set of biomarkers and multiomics approach, offers a promising tool for early detection. GPs can leverage this test to identify physiological harm, guide interventions, and report to CPS when needed. Legal acceptance of parental alienation varies, influencing the test’s adoption, with potential to support custody decisions where recognized. Longitudinal studies are crucial to validate the diagnostic test and enhance its integration into clinical and legal frameworks.

## Supplementary Information

Below is the link to the electronic supplementary material.

Supplementary Material 1

## Data Availability

All data generated or analyzed during this study are included in this published article and its supplementary information files.
