# Supplementary material for: Towards molecular diagnostics of parental alienation
Source: Cell Mol Life Sci. 2025 Nov 6;82(1):383. doi: 10.1007/s00018-025-05895-3 (PMC12592572; doi:10.1007/s00018-025-05895-3)
Supplement: Supplementary file 1 — Supplementary Material 1 [file 18_2025_5895_MOESM1_ESM.docx]

Table S1: Interventions Targeting Molecular Mechanisms

| **Molecular Mechanism** | **Pediatric-Specific Intervention** | **Targeted Health Outcome** | **Mechanistic Rationale** | **Evidence Base and Considerations** | **Expected Pediatric Outcome** |
| --- | --- | --- | --- | --- | --- |
| **HPA Axis Dysregulation** | **Cognitive Behavioral Therapy (CBT) tailored for children, mindfulness-based stress reduction (MBSR) programs (e.g., Mindful Schools), low-dose hydrocortisone modulation (under strict pediatric endocrinology supervision)** | Eating disorders, T1D, endocrine disruptions | Normalizes cortisol secretion by reducing CRH and ACTH hypersecretion; enhances stress coping via neuroplasticity in the prefrontal cortex. | CBT reduces HPA axis hyperactivity in children (Murphy et al., 2022; Lange et al., 2025) (1, 2). MBSR shows efficacy in reducing cortisol in adolescents (Bloch et al., 2007; Hue et al., 2025) (3, 4). Hydrocortisone requires careful monitoring due to growth suppression risks in children. | Reduced cortisol levels, improved appetite regulation, stabilized glucose metabolism, and enhanced emotional resilience in children. |
| **Neurotransmitter Imbalances** | **Pediatric-specific SSRIs (e.g., fluoxetine, FDA-approved for children >8 years), play therapy, omega-3 fatty acid supplementation (DHA/EPA)** | Depression, anxiety, eating disorders | Restores serotonin and dopamine homeostasis; play therapy enhances emotional expression and dopaminergic reward pathways; omega-3 supports neuronal membrane integrity. | SSRIs improve pediatric depression (Belujon & Grace, 2017; Dwyer & Bloch, 2019) (5, 6); play therapy aids emotional regulation (Martin & Ochsner, 2016; Gupta et al., 2023) (7, 8); omega-3 reduces depressive symptoms in children (Girotti et al., 2024; Li et al., 2024) (9, 10). Monitor SSRI side effects (e.g., agitation). | Improved mood stability, reduced anxiety, and normalized eating behaviors in children. |
| **Inflammation** | **Anti-inflammatory diet (e.g., Mediterranean diet adapted for children), NSAIDs (e.g., ibuprofen, short-term use), stress-reduction programs (e.g., school-based yoga)** | Immune dysfunction, allergic reactions, cardiovascular risks | Reduces pro-inflammatory cytokines (IL-6, TNF-α, CRP); dietary interventions enhance gut-derived anti-inflammatory metabolites; yoga modulates vagal tone. | Mediterranean diet lowers IL-6 in children (Barakat et al., 2023; Fuentes-Albero et al., 2024) (11, 12); yoga reduces inflammatory markers in adolescents (Konstantinou et al., 2022; Estevao, 2022) (13, 14). NSAIDs require caution due to pediatric gastrointestinal risks. | Decreased systemic inflammation, reduced allergy exacerbations, and lower cardiovascular risk factors in children. |
| **Oxidative Stress** | **Antioxidant supplementation (e.g., vitamin C, alpha-lipoic acid, pediatric-safe doses), N-acetylcysteine (NAC), environmental enrichment programs** | Cardiovascular damage, neurological impairments | Neutralizes ROS, enhances glutathione synthesis; environmental enrichment promotes neuroprotection via BDNF upregulation. | NAC reduces oxidative stress in pediatric populations (Halabitska et al., 2024; Babu et al., 2024) (15, 16); enrichment programs improve cognitive outcomes (Kappou et al., 2021; Coleman et al., 2023) (17, 18). Avoid high-dose antioxidants to prevent pro-oxidant effects in children. | Reduced cellular damage, improved cognitive function, and enhanced neuroprotection in children. |
| **Epigenetic Modifications** | **Trauma-focused CBT, folate supplementation (methyl donor), histone deacetylase (HDAC) inhibitors (investigational, e.g., valproate)** | Mood disorders, endocrine disruptions | Reverses stress-induced DNA methylation (e.g., NR3C1 gene); folate supports methyl metabolism; HDAC inhibitors restore gene expression profiles. | Trauma-focused CBT reduces NR3C1 methylation in children (Dieckmann & Czamara, 2024; Parade et al., 2021) (19, 20); folate aids epigenetic stability (Loh et al., 2024; Michels et al., 2024) (21, 22). HDAC inhibitors are experimental and require pediatric trials. | Improved stress resilience, normalized gene expression, and reduced long-term mood disorder risk in children. |
| **Gut Microbiota Dysbiosis** | **Pediatric probiotics (e.g., Lactobacillus rhamnosus GG), prebiotics (e.g., inulin), gut-brain axis-focused dietary counseling** | Gastrointestinal disorders, mood disorders | Restores microbiota diversity, increases SCFA production, enhances gut barrier integrity; dietary counseling promotes fiber-rich diets suitable for children. | Probiotics improve IBS symptoms in children (Ramadan et al., 2025; Kianifar et al., 2015) (23, 24); prebiotics enhance mood via gut-brain axis (O'Riordan et al., 2025; Ansari et al., 2023) (25, 26). Monitor for bloating in sensitive pediatric populations. | Improved gut health, reduced IBS symptoms, and enhanced mood stability in children. |
| **Autonomic Nervous System Imbalance** | **Biofeedback training (e.g., heart rate variability biofeedback), child-friendly relaxation techniques (e.g., guided imagery), vagal nerve stimulation (non-invasive, investigational)** | Cardiovascular issues, gastrointestinal disorders | Restores sympathetic-parasympathetic balance; enhances vagal tone to regulate heart rate and gut motility. | Biofeedback improves autonomic regulation in children (Gruescu et al., 2024; Aritzeta et al., 2022) (27, 28); guided imagery reduces stress responses (Barbieri et al., 2023; Zemla et al., 2023) (29, 30). Vagal stimulation requires further pediatric validation. | Normalized heart rate variability, reduced gastrointestinal symptoms, and improved stress coping in children. |
| **Mitochondrial Dysfunction** | **Coenzyme Q10 supplementation, mitochondrial-targeted antioxidants (e.g., MitoQ, pediatric-adjusted doses), structured physical activity programs** | Neurological impairments, fatigue | Enhances mitochondrial ATP production, reduces ROS-induced mitochondrial damage; physical activity upregulates mitochondrial biogenesis. | CoQ10 improves fatigue in pediatric populations (Di Carlo & Sorrentino, 2024; Tsai et al., 2022) (31, 32); physical activity enhances neurodevelopment (Mbiydzenyuy & Qulu, 2024; Jing et al., 2024) (33, 34). Monitor for gastrointestinal side effects. | Reduced fatigue, improved cognitive function, and enhanced neurodevelopmental outcomes in children. |
| **Telomere Shortening** | **Lifestyle interventions (e.g., sleep hygiene, physical activity), antioxidant-rich diet, stress-reduction programs (e.g., family therapy)** | Cardiovascular disease, premature aging | Slows telomere attrition by reducing oxidative stress and inflammation; family therapy mitigates chronic stress exposure. | Sleep and exercise preserve telomere length in children (Huang et al., 2025; Sabot et al., 2023) (35, 36); family therapy reduces stress (Melton & McLeigh, 2020; Chen et al., 2021) (37, 38). Long-term studies needed for pediatric outcomes. | Slowed biological aging, reduced cardiovascular risk, and improved long-term health in children. |
| **Neuroplasticity Impairment** | **Neurofeedback training, enriched environments (e.g., music/art therapy), cognitive training programs (e.g., working memory exercises)** | Cognitive deficits, emotional dysregulation | Enhances synaptic plasticity in hippocampus/prefrontal cortex; promotes BDNF expression and neural connectivity. | Neurofeedback improves attention in children (Holt-Gosselin et al., 2024; Holtmann et al., 2014) (39, 40); enriched environments enhance neuroplasticity (Kappou et al., 2021; Han et al., 2022) (17, 41). Requires consistent engagement for efficacy. | Improved cognitive performance, enhanced emotional regulation, and reduced learning difficulties in children. |

References

1. Murphy F, Nasa A, Cullinane D, Raajakesary K, Gazzaz A, Sooknarine V, et al. Childhood Trauma, the HPA Axis and Psychiatric Illnesses: A Targeted Literature Synthesis. Frontiers in psychiatry. 2022;13:748372.

2. Lange J, Erhardt-Lehmann A. HPA system in anxiety disorder patients treated with cognitive behavioural therapy: A review. Biomarkers in Neuropsychiatry. 2025;12:100116.

3. Bloch MH, Landeros-Weisenberger A, Dombrowski P, Kelmendi B, Wegner R, Nudel J, et al. Systematic review: pharmacological and behavioral treatment for trichotillomania. Biological psychiatry. 2007;62(8):839-46.

4. Hue VC, Siaw Y-L, Mohamad Nor A. A systematic review of mindfulness-based stress reduction in the management of anxiety disorders among adolescents and young adults aged 13–26. Asian Journal of Psychiatry. 2025;108:104497.

5. Belujon P, Grace AA. Dopamine System Dysregulation in Major Depressive Disorders. The international journal of neuropsychopharmacology. 2017;20(12):1036-46.

6. Dwyer JB, Bloch MH. Antidepressants for Pediatric Patients. Current psychiatry. 2019;18(9):26-42f.

7. Martin R, Ochsner K. The Neuroscience of Emotion Regulation Development: Implications for Education. Current opinion in behavioral sciences. 2016;10.

8. Gupta N, Chaudhary R, Gupta M, Ikehara LH, Zubiar F, Madabushi JS. Play Therapy As Effective Options for School-Age Children With Emotional and Behavioral Problems: A Case Series. Cureus. 2023;15(6):e40093.

9. Girotti M, Bulin SE, Carreno FR. Effects of chronic stress on cognitive function - From neurobiology to intervention. Neurobiology of stress. 2024;33:100670.

10. Li S, Li R, Hu X, Zhang Y, Wang D, Gao Y, et al. Omega-3 supplementation improves depressive symptoms, cognitive function and niacin skin flushing response in adolescent depression: A randomized controlled clinical trial. Journal of Affective Disorders. 2024;345:394-403.

11. Barakat S, McLean SA, Bryant E, Le A, Marks P, Touyz S, et al. Risk factors for eating disorders: findings from a rapid review. Journal of eating disorders. 2023;11(1):8.

12. Fuentes-Albero M, Mafla-España MA, Martínez-Raga J, Cauli O. Autistic Children/Adolescents Have Lower Adherence to the Mediterranean Diet and Higher Salivary IL-6 Concentration: Potential Diet–Inflammation Links? Pathophysiology : the official journal of the International Society for Pathophysiology. 2024;31(3):376-87.

13. Konstantinou GN, Konstantinou GN, Koulias C, Petalas K, Makris M. Further Understanding of Neuro-Immune Interactions in Allergy: Implications in Pathophysiology and Role in Disease Progression. Journal of asthma and allergy. 2022;15:1273-91.

14. Estevao C. The role of yoga in inflammatory markers. Brain, behavior, & immunity - health. 2022;20:100421.

15. Halabitska I, Oksenych V, Kamyshnyi O. Exploring the Efficacy of Alpha-Lipoic Acid in Comorbid Osteoarthritis and Type 2 Diabetes Mellitus2024.

16. Babu Balagopal P, Kohli R, Uppal V, Averill L, Shah C, McGoogan K, et al. Effect of N-acetyl cysteine in children with metabolic dysfunction-associated steatotic liver disease-A pilot study. Journal of pediatric gastroenterology and nutrition. 2024;79(3):652-60.

17. Kappou K, Ntougia M, Kourtesi A, Panagouli E, Vlachopapadopoulou E, Michalacos S, et al. Neuroimaging Findings in Adolescents and Young Adults with Anorexia Nervosa: A Systematic Review. Children (Basel, Switzerland). 2021;8(2).

18. Coleman ME, Roessler MEH, Peng S, Roth AR, Risacher SL, Saykin AJ, et al. Social enrichment on the job: Complex work with people improves episodic memory, promotes brain reserve, and reduces the risk of dementia. Alzheimer's & dementia : the journal of the Alzheimer's Association. 2023;19(6):2655-65.

19. Dieckmann L, Czamara D. Epigenetics of prenatal stress in humans: the current research landscape. Clinical Epigenetics. 2024;16(1):20.

20. Parade SH, Huffhines L, Daniels TE, Stroud LR, Nugent NR, Tyrka AR. A systematic review of childhood maltreatment and DNA methylation: candidate gene and epigenome-wide approaches. Translational Psychiatry. 2021;11(1):134.

21. Loh JS, Mak WQ, Tan LKS, Ng CX, Chan HH, Yeow SH, et al. Microbiota–gut–brain axis and its therapeutic applications in neurodegenerative diseases. Signal transduction and targeted therapy. 2024;9(1):37.

22. Michels KB, Binder AM. Impact of folic acid supplementation on the epigenetic profile in healthy unfortified individuals - a randomized intervention trial. Epigenetics. 2024;19(1):2293410.

23. Ramadan YN, Alqifari SF, Alshehri K, Alhowiti A, Mirghani H, Alrasheed T, et al. Microbiome Gut-Brain-Axis: Impact on Brain Development and Mental Health. Molecular Neurobiology. 2025.

24. Kianifar H, Jafari SA, Kiani M, Ahanchian H, Ghasemi SV, Grover Z, et al. Probiotic for irritable bowel syndrome in pediatric patients: a randomized controlled clinical trial. Electronic physician. 2015;7(5):1255-60.

25. O'Riordan KJ, Moloney GM, Keane L, Clarke G, Cryan JF. The gut microbiota-immune-brain axis: Therapeutic implications. Cell reports Medicine. 2025;6(3):101982.

26. Ansari F, Neshat M, Pourjafar H, Jafari SM, Samakkhah SA, Mirzakhani E. The role of probiotics and prebiotics in modulating of the gut-brain axis. Frontiers in nutrition. 2023;10:1173660.

27. Gruescu ACS, Popoiu C, Levai MC, Barata PI, Streian CG. Evaluating Family Coping Mechanisms in Pediatric Seizure Disorders: From Emergency Room to Long-Term Follow-Up. Pediatric reports. 2024;16(3):657-68.

28. Aritzeta A, Aranberri-Ruiz A, Soroa G, Mindeguia R, Olarza A. Emotional Self-Regulation in Primary Education: A Heart Rate-Variability Biofeedback Intervention Programme. International journal of environmental research and public health. 2022;19(9).

29. Barbieri V, Plagg B, Marino P, Piccoliori G, Engl A. Fortifying the Foundations: A Comprehensive Approach to Enhancing Mental Health Support in Educational Policies Amidst Crises. Healthcare. 2023;11:1423.

30. Zemla K, Sedek G, Wróbel K, Postepski F, Wojcik GM. Investigating the Impact of Guided Imagery on Stress, Brain Functions, and Attention: A Randomized Trial. Sensors (Basel, Switzerland). 2023;23(13).

31. Di Carlo E, Sorrentino C. Oxidative Stress and Age-Related Tumors. Antioxidants. 2024;13(9):1109.

32. Tsai IC, Hsu CW, Chang CH, Tseng PT, Chang KV. Effectiveness of Coenzyme Q10 Supplementation for Reducing Fatigue: A Systematic Review and Meta-Analysis of Randomized Controlled Trials. Frontiers in pharmacology. 2022;13:883251.

33. Mbiydzenyuy NE, Qulu L-A. Stress, hypothalamic-pituitary-adrenal axis, hypothalamic-pituitary-gonadal axis, and aggression. Metabolic Brain Disease. 2024;39(8):1613-36.

34. Jing J-Q, Jia S-J, Yang C-J. Physical activity promotes brain development through serotonin during early childhood. Neuroscience. 2024;554:34-42.

35. Huang X, Huang L, Lu J, Cheng L, Wu D, Li L, et al. The relationship between telomere length and aging-related diseases. Clinical and Experimental Medicine. 2025;25(1):72.

36. Sabot D, Lovegrove R, Stapleton P. The association between sleep quality and telomere length: A systematic literature review. Brain, behavior, & immunity - health. 2023;28:100577.

37. Melton GB, McLeigh JD. The Nature, Logic, and Significance of Strong Communities for Children. International journal on child maltreatment : research, policy and practice. 2020;3(2):125-61.

38. Chen Q, Zhao W, Li Q, Sagi H. The influence of family therapy on psychological stress and social adaptability of depressed patients. Work (Reading, Mass). 2021;69(2):613-24.

39. Holt-Gosselin B, Keding TJ, Rodrigues K, Rueter A, Hendrickson TJ, Perrone A, et al. Familial risk for depression moderates neural circuitry in healthy preadolescents to predict adolescent depression symptoms in the Adolescent Brain Cognitive Development (ABCD) Study. Developmental Cognitive Neuroscience. 2024;68:101400.

40. Holtmann M, Pniewski B, Wachtlin D, Wörz S, Strehl U. Neurofeedback in children with attention-deficit/hyperactivity disorder (ADHD)--a controlled multicenter study of a non-pharmacological treatment approach. BMC pediatrics. 2014;14:202.

41. Han Y, Yuan M, Guo YS, Shen XY, Gao ZK, Bi X. The role of enriched environment in neural development and repair. Frontiers in cellular neuroscience. 2022;16:890666.
